# Reliable electricity to advance quality health care in low-resource settings

**DOI:** 10.1016/S2214-109X(25)00537-6

**Published:** 2026-03-16

**Authors:** Salvatore Vinci, Laura H Kwong, Samuel B Miles, Ryan McCord, Gina Cady, Kun Tang, Wenlu Ye, Harish Hande, Bineta Mbacke, Heather Adair-Rohani, Thomas Clasen, Daniel M Kammen

**Affiliations:** a World Health Organization, Geneva, Switzerland; b Division of Environmental Health Sciences, School of Public Health, University of California, Berkeley, Berkeley, CA, USA; c Department of Civil and Systems Engineering, Johns Hopkins University, Baltimore, MD, USA; d Sanford School of Public Policy, Nicholas School of the Environment, Duke University, Durham, NC, USA; e Health Electrification and Telecommunications Alliance, USAID, Washington, DC, USA; f Vanke School of Public Health, Tsinghua University, Beijing, China; g SELCO Foundation, Bengaluru, Karnataka, India; h Strategic Innovation & New Investors, Gavi, the Vaccine Alliance, Geneva, Switzerland; i Gangarosa Department of Environmental Health, Rollins School of Public Health, Emory University, Atlanta, GA, USA; j Ralph O’Connor Sustainable Energy Institute, Johns Hopkins University, Baltimore, MD, USA; k Paul Nitze School of Advanced International Studies, Johns Hopkins University, Baltimore, MD, USA

## Abstract

Reliable, affordable, sustainable, and equitable electricity access in health-care facilities is fundamental to achieving universal health coverage. Decentralised renewable energy systems coupled with battery storage present a key opportunity to rapidly expand access to reliable electricity, particularly for remote facilities where grid connection is unavailable and as a backup for facilities with unreliable power. In this Series paper, we discuss several measures and policy options for accelerating health-care facility electrification, including strengthening monitoring and standardising indicators for electricity services at health-care facilities, prioritising and increasing equitable investment in energy infrastructure for health-care facilities, strengthening coordination between health, energy, and other sectors, building local capacity to instal, operate, and maintain energy systems at health-care facilities, and developing and disseminating energy-efficient biomedical equipment suitable for harsh conditions. Innovative cooperation approaches that leverage synergies between different partners can maximise effect, such as by expanding cold chain platforms to cover all energy needs of health facilities. We highlight effective approaches to ensure long-term functionality of solar electricity systems through tailored delivery models and operation and maintenance arrangements, as well as the need for integrating health-care facility electrification into overall health system strengthening strategies. This integration would also ensure that adequate data are gathered and made available, which is essential to increase research and evaluate outcomes of health-care facility electrification interventions. Achieving universal access to reliable electricity in health-care facilities is both a moral imperative and a strategic investment that will pay dividends in improved health outcomes for all.

This is the fourth in a **Series** of five papers on energy and health in low-income and middle-income countries. All papers in the Series are available at www.thelancet.com/series-do/energy-health-2026

## Introduction

In health-care facilities without electricity, providers are forced to deliver care under extremely constrained conditions, relying on torches, candles, and kerosene lamps for lighting; using wood or coal to boil water and sterilise instruments;^1^ or relying on manual bagging for oxygen provision. These coping strategies highlight that electricity is foundational for quality health services. It powers essential infrastructure such as lighting, water pumps, cold-chain systems, equipment for safe deliveries and neonatal care, electronic health records, and telemedicine. Electricity also enables the operation of critical medical devices—including oxygen concentrators, ventilators, neonatal incubators, and defibrillators—and supports laboratory and diagnostic technologies, such as x-rays and ultrasound machines. Beyond clinical functions, reliable power improves the safety and security of patients and staff, strengthens morale and retention of staff, and reinforces community trust in the health system.^2^

Although some procedures can be done with non-electric alternatives, many modern diagnostics and life-saving interventions are not possible without electricity. Consequently, the availability and reliability of a power supply play a key role in enabling broader, quality health services, defined as health services that are effective, safe, people-centred, timely, equitable, integrated, and efficient.^3^ Reliable access to energy is also crucial to enable coordination and response during outbreaks of disease and pandemics. Before the Ebola virus disease outbreak in 2014, several operational medical facilities lacked potable water, lighting, equipment, and refrigeration.^4^ During the COVID-19 pandemics, oxygen and vaccine delivery were stunted where electricity supplies and refrigeration were unstable or absent.^5^

Even when electricity is available, it can fail to meet health-care facility needs when it is unreliable, of low-quality, or otherwise insufficient. Unreliable electricity can interrupt critical health services, damage essential equipment, and delay or prevent testing, contact tracing, vaccination campaigns, and communication, including in cases of emergency referrals.

Recognising the urgency of providing all health-care facilities with reliable electricity and with water, sanitation, hygiene, and waste services, the UN General Assembly unanimously adopted the resolution “sustainable, safe and universal water, sanitation, hygiene, waste, and electricity services in health-care facilities” in 2023.^6^ The resolution calls for accelerated action and investments, international cooperation, cross-sectoral engagement, resource mobilisations, and regular monitoring and evaluation.

In this paper, the fourth in the Series on energy and health in low-income and middle-income countries, we aim to underscore the essential role of reliable electricity in enabling quality health care, describe how decentralised renewable energy can rapidly close critical power gaps in low-resource settings, while outlining key policy, financing, and coordination strategies that can accelerate action and translate electrification into sustained gains in service delivery and health outcomes.

**Key messages**

• Reliable electricity is essential to enable quality health services, powering life-saving devices and critical appliances, diagnostics, digital health, and emergency response capacities
• About 1 billion people in low-income and lower-middle-income countries are served by health-care facilities with no or unreliable electricity, undermining safe, timely, and effective health services
• Decentralised renewable energy solutions, such as solar-plus-battery systems, represent a rapidly deployable, cost-effective, and climate-resilient opportunity to electrify health-care facilities, particularly in remote or underserved areas
• Long-term operation and maintenance of solar systems is crucial to ensure system functionality, requiring sustained financing and clear roles and responsibilities
• Accelerating electrification requires increased investments and coordinated policy action, including cross-sectoral collaboration, climate-finance integration, standardised indicators, and equitable prioritisation of underserved communities
• Electricity access interventions must be paired with power-dependent medical devices, trained staff, and broader health-system strengthening efforts to ensure that electrification translates into real improvements in service delivery and health outcomes

## Health facility electrification, service delivery, and health and economic outcomes

**Figure 1:**
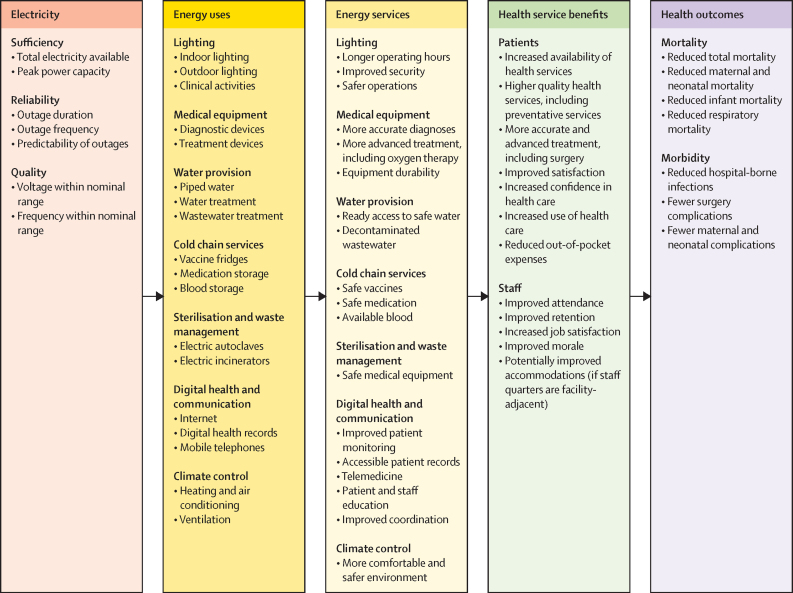
Potential pathways and contribution of electricity to service delivery and health outcomes

Although the link between reliable electricity and proper functioning of power-dependent medical devices is clear, empirical evidence on the effects of health facility electrification on service delivery and health and economic outcomes is relatively scarce. As such, this paper uses illustrative examples from completed and ongoing projects to supplement the academic literature. Figure 1 provides a visualisation of the potential links between electricity and health services and the technical and human pathways through which electricity can improve service delivery and, in turn, health outcomes.

Emerging evidence increasingly links facility-level electrification to improved health service delivery. In Uganda, a cluster-randomised trial revealed that installation of solar-powered lighting in 30 maternity facilities increased the quality of care and reduced delays.^7^ In Gujarat, India, a rural electrification programme improved health facility functionality and increased service use, by enabling the use of a wide range of essential devices and equipment, such as deep freezers, ice-lined refrigerators, cold boxes, and vaccine carriers.^8^ Synthesising evidence across low-income and middle-income countries (LMICs), a 2022 systematic review concluded that electrification of health-care facilities was associated with improvements in the quality of antenatal care services, vaccination rates, emergency capabilities, and primary health services, with many facilities reporting high-quality, reliable, and continuous oxygen supplies, refrigeration, and enhanced medical supply chains.^5^

Although the effect of facility-level electrification on health outcomes has not been thoroughly studied experimentally, a study conducted in 2016 in Ghana revealed a positive association between the frequency of power outages and in-facility mortality, with the risk of mortality estimated to increase by 43% for each day the power was out for more than 2 h.^9^ In Papua New Guinea, the use of solar-powered oxygen concentrators in 38 remote health facilities that otherwise could not provide medical oxygen was associated with a decreased incidence of pneumonia deaths from 2·83 to 1·17 per 100 pneumonia admissions.^10^

In terms of value for money, in Uganda, a randomised controlled trial on oxygen delivery methods for patients with pneumonia found that the cost per disability-adjusted life-year saved was US$27 for a solar oxygen concentrator and $50 for cylinder oxygen.^11^ In Papua New Guinea, the incremental cost-effectiveness ratio was $50 per disability-adjusted life-year averted (95% CI $39–70), $1673 per life saved (95% CI $1281–2317), and $51 per patient treated.^12^ Similarly, a modelled incremental cost-effectiveness ratio of solar oxygen concentrators compared with no oxygen was $20 per disability-adjusted life-year saved (95% CI $2·83–206·00), $542 per life saved, and $26 per patient treated.^13^

Although device-specific solarisation interventions are helpful to illustrate the health effect of solarisation, and could be used in critical situations, the goal of electrification interventions should be to provide reliable, high-quality power for all essential services within a facility, whenever possible, rather than using a siloed approach. WHO estimates that providing just 30 000 unelectrified primary health-care facilities with reliable electricity and essential medical devices could save up to 7·84 million lives by 2050.^14^ Country-focused analyses developed with UNICEF support estimate that improving access to resilient energy in health-care facilities could avert over 175 000 deaths in Pakistan and almost 111 000 deaths in Tanzania from 2024 to 2030.^15^ With the effect on health in terms of higher immunisation rates and a reduced burden of disease, this increased access could contribute an estimated $296 million to the economy of Pakistan compared with an investment cost of $128·1 million and $360 million to Tanzania compared with an investment cost of $170·5 million, by 2044.

The World Bank estimates that providing all health-care facilities in 63 LMICs with new connections (for unelectrified facilities) or backup systems (in facilities with unreliable supply) would cost $4·9 billion.^2^ The resources announced for energy interventions during the Africa Energy Summit in February 2025 were about ten times this amount.^16^ However, health-care facility electrification has not yet been prioritised when allocating renewable energy and climate change funds.

## Status of electricity access in health-care facilities

Data on electricity access in health-care facilities are often scarce or outdated. These data are rarely collected systematically, are often limited to specific projects or regional aggregations, and are typically estimated rather than measured directly. Key measurement platforms include the database on electrification of health-care facilities, managed by WHO, which gathers and analyses data on electrification status, and the Global Heatmap of health facility electrification—with the accompanying database—which is managed by Sustainable Energy for All and gathers and analyses data on key electrification programmes, with the aim to provide unified and comprehensive information.^17^, ^18^

Current estimates indicate that nearly 1 billion people in low-income and lower-middle-income countries are served by health-care facilities without reliable electricity access (about 478 million) or with no electricity access at all (about 433 million).^2^ In low-income and lower-middle-income countries of sub-Saharan Africa and south Asia, at least 15% (sub-Saharan Africa) and 12% (south Asia) of health-care facilities, are estimated to have no electricity access, with lower rates of access in rural than in urban areas.^2^ Furthermore, only half of hospitals in sub-Saharan Africa are estimated to have access to reliable electricity.^2^

Low-quality electricity (electricity that fails to conform to the necessary characteristics, principally in terms of voltage and frequency) can also cause malfunction, accelerated degradation, and failure of medical equipment. It is estimated that 40–70% of medical devices and equipment in LMICs are broken, unused, or unfit for purpose,^19^ with low-quality electricity being one of the main causes of medical device failures.^20^ Under-voltage electricity supply might support only low-draw devices such as lightbulbs, whereas over-voltage can short-circuit expensive equipment such as x-ray machines, causing permanent damage or even electrical fires. Alternating current frequency deviations, although less common, can affect electrical motor speeds, potentially interfering with sensitive diagnostic tests such as blood centrifugation, precision dosing, or medical imaging. Table 1 illustrates examples of selected power quality and reliability metrics used in different countries.

**Table 1:** Illustrative examples of selected power quality and reliability metrics measured at health facilities in three countries^21^, ^22^

	**Nigeria (n=93 health facilities over 18 months)**	**Democratic Republic of the Congo (n=25 health facilities over 29 months)**	**Kenya (n=210 health facilities over 18 months)**
Voltage within 10% of nominal	27·0%	73·9%*	87·0%
Frequency within 10% of nominal	97·8%	84·9%*	99·9%
Interruption incidence, mean (SD), outages per day	2·05	0·67 (0·53)	1·33
Interruption duration, median (IQR)†	6·0 min	13·1 min (6·4–81·0)	1·2 min

* For the Democratic Republic of the Congo, percentages are calculated as n/N, where n is the number of ~2-minute sensor readings within range and N is the total number of readings used in the analysis (voltage: 16 065 593/21 743 671; frequency: 18 469 681/21 743 671).

† Interruption duration is defined as the length of individual outage events. Median and IQR are reported where raw data were available; for Nigeria and Kenya, values are reproduced from published sources; underlying sample-level counts were not reported.

Although the effects of short-duration power interruptions can be mitigated through battery storage or uninterrupted power supply devices connected to critical equipment, longer interruptions necessitate that facilities rely on secondary energy sources. Diesel-fuelled generators and photovoltaic systems are some of the systems typically used as backup power solutions in grid-connected facilities, or as main power supply sources for facilities in off-grid areas.^23^, ^24^ Facilities use diesel fuel generators due to their wide availability, low up-front costs, and use readiness. However, diesel fuel costs over time and disruptions in the fuel supply chain—for example, due to conflict, extreme weather, and geopolitical tensions—represent substantial challenges,^25^, ^26^ which make electricity from diesel-fuelled generators less reliable and, in the medium term and long term, more expensive than electricity from solar systems,^27^ particularly in remote and rural areas. Combustion of diesel fuel also generates air pollution and noise close to health facilities, in addition to increased carbon emissions.^28^

In some contexts, conflict, organised violence, and insecurity can disrupt grid-connected and off-grid power supplies. In such fragile environments, it becomes crucial to adapt power system planning to account for security risks, including theft of equipment (particularly copper wiring and solar modules) or diesel fuel, which can jeopardise the electricity supply to health centres. This planning is especially crucial for decentralised systems in remote areas, or where there is minimal security and replacement parts can be costly and difficult to procure.^29^

## Opportunities to accelerate electrification of health-care facilities

### Electricity requirements and supply options for health-care facilities

Energy needs in health-care facilities vary according to factors such as location, size, geography, catchment population, operating hours, and the specific health services offered. There is no one-size-fits-all solution to meeting facility-specific energy requirements; however, these factors can be used to define targeted power-supply benchmarks that optimise both resource allocation and service quality.^2^, ^30^, ^31^

Primary health-care facilities, which provide essential services such as vaccinations, deliveries, and preventive care, usually require a stable power supply to enable access to clean water, adequate lighting, selected life-saving medical devices, laboratory equipment, and sterilisation. Depending on the specific contexts, these facilities might require less than 20 kWh per day,^2^ whereas larger referral hospitals might require several hundred kWh per day. Even within a single facility, energy demands can vary substantially across wards. Critical areas such as delivery rooms, intensive care units, and surgical wards require uninterrupted power to ensure a high standard of care and to prevent avoidable deaths. In settings with inadequate energy availability, prioritisation of these critical services might be necessary. For example, facilities can designate specific services or wards as critical and configure electrical systems to ensure uninterrupted power through dedicated backup supply systems.

Expanding the electricity grid can be viable for some health-care facilities, particularly large hospitals with substantial demand or near a reliable grid. However, grid extension can be a lengthy process in some contexts, and numerous health facilities in low-income settings are far from the grid and therefore require off-grid solutions able to address energy challenges in an effective and timely manner. The decreasing costs of decentralised renewable energy—in particular of solar-plus-battery solutions—coupled with long-term operation and maintenance arrangements, offer opportunities for sustainably addressing the electricity needs of health-care facilities, as a main energy source or as a backup system.

Over the past decade, several countries have advanced health-care facility electrification through investments in decentralised renewable energy—particularly standalone solar systems—and, in some cases, have coordinated these electrification efforts with the provision of power-dependent medical devices, such as in Nepal.^2^ In addition to standalone solar systems tailored to individual facilities, mini-grids powered by renewable energy can also represent an effective approach for electrifying multiple health-care facilities and other buildings, such as schools, in areas not reached by the central grid. Planning power systems with the integrated goals of health-care facility electrification and community development offers an opportunity to cost-effectively advance multiple national priorities simultaneously and to maximise socioeconomic impact.^32^

Despite substantial progress in past decade, several key barriers continue to slow the scale-up of health-care facility electrification, including insufficient financial support, inadequate long-term operation and maintenance arrangements for solar systems, and poor coordination among key actors.

### Decentralised, renewable energy systems to accelerate electrification, increase climate resilience, and reduce dependence on fossil fuels

Decentralised renewable energy systems, such as photovoltaic modules coupled with battery storage, present a major opportunity to rapidly deploy reliable, clean, and climate-resilient electricity to health-care facilities, including in remote locations, without waiting for grid extension or upgrades or relying on diesel-fuelled generators. Solar-plus-battery systems offer a readily deployable solution that can be customised to meet the specific energy needs of individual facilities; they can function as the primary energy source in off-grid settings or as a backup solution where grid power is unreliable.^33^ A further advantage of solar-plus-battery systems is their modularity, which allows systems to be tailored and scaled to diverse energy needs and expanded relatively easily as demand increases.

An innovative example of multipartner cooperation that leverages these characteristics is the partnership between WHO, Gavi, the Vaccine Alliance, and UNICEF, aiming to provide electricity to health-care facilities, building on the cold chain equipment optimisation platform (CCEOP).^34^ Since the main objective of the CCEOP is to guarantee adequate vaccine preservation, the platform has traditionally focused on supporting the deployment of solar systems for vaccine refrigeration, and in particular, the deployment of solar direct drive refrigerators in unelectrified facilities. In consideration of the decreasing costs and the modularity of solar systems and the effect of enabling multiple health services in addition to immunisation, the CCEOP approach has been revised in four pilot countries, starting in 2023. Instead of supporting the deployment of solar systems that meet only the energy needs of vaccine refrigeration, the scope has been expanded to cover all energy requirements of about 1300 primary health-care facilities across Zambia, Uganda, Ethiopia, and Pakistan. This expansion will have multiple effects on health by addressing health needs in a holistic way. Furthermore, the initiative also covers the basic energy loads of staff quarters at the facilities, such as lighting and communication, thereby enabling better living conditions.

With the sharply declining costs of photovoltaic modules and batteries in the past two decades, solar-based electricity has become less expensive than diesel-fuel-based electricity in most areas. Prices of solar systems might vary substantially according to different country contexts but, as examples, the prices of 5 kW solar systems coupled with 12 kWh batteries recently installed in primary health-care facilities in selected countries in sub-Saharan Africa have ranged between $10 000 and $19 000, with an extra cost of about $400 to $500 per facility per year for operation and maintenance for the initial 5 years.

The International Renewable Energy Agency estimates that, in Mali, transitioning from diesel-based solutions to solar energy would allow a payback of the initial investment in approximately 2·0–6·5 years, depending on the amount of diesel consumption.^35^ According to Sustainable Energy for All, the approximate annual cost savings of installing solar photovoltaic systems in energy-deficient health-care facilities would be $125–207 million in India, $34–36 million in Nigeria, and $2·9–5·1 million in Nepal.^36^

The speed of installation and independence from diesel fuel also makes decentralised solar power systems particularly suitable for emergency and humanitarian contexts. Recognising this potential, WHO and UNICEF, with support from SELCO Foundation, have integrated rapidly deployable solar systems within the prototypes of an infectious disease treatment module and a health emergency facility for use in outbreak response and other emergency contexts.^37^, ^38^ A study of power in humanitarian health-care facilities, which commonly rely solely on diesel power, estimated that transitioning from 11 365 diesel generators to solar energy would reduce electricity costs by two-thirds, saving $70 million per year, while also reducing greenhouse gas emissions by 126 000 tonnes of CO_2_-equivalent.^27^

### Ensuring long-term maintenance for health facility energy systems

Long-term operation and maintenance of health facility energy systems, including for decentralised solar solutions, is crucial for sustained provision of the delivery of essential health services. Although systematic data on failure rates are scarce, experience indicates that the instal-and-forget approach used in several health-care facility electrification programmes leads to dysfunctional solar systems within a few years of installation. Key challenges include unclear roles and responsibilities, insufficient financing for long-term operation and maintenance contracts or for the replacement of batteries and spare parts, insufficient access to skilled technicians, and poor accountability for system failures.^39^

For decentralised solar systems coupled with batteries, operation and maintenance refers to preventive, scheduled, and corrective maintenance—activities that are often ignored in long-term planning and budget allocation decisions. Annual operation and maintenance costs for decentralised solar systems typically range from 3% to 5% of initial equipment costs, and battery replacements every 5–7 years can cost up to 35% of the initial investment.^2^ Replacing lead acid batteries with lithium-ion chemistries, for example, offers the opportunity to lower lifetime environmental costs.^40^

In several donor-funded programmes in low-resource settings, donor budgets typically cover the initial capital costs for equipment purchase and installation, but too often no dedicated allocation is made for long-term operation and maintenance, with the expectation that a third party—such as the Ministry of Health or the health facility itself—will cover these costs. Several models are being explored to cover long-term operation and maintenance costs in low-resource settings, from long-term operation and maintenance contracts with local actors to energy service-based models, however, the lack of dedicated funding remains a key challenge. Remote monitoring can further support functionality of the systems by enhancing efficiency and transparency, facilitating early identification of faults, and reducing travel and response times for troubleshooting. Remote monitoring platforms can also be used to trigger performance-based payments and to enable access to complementary support mechanisms, such as carbon credits.

In some settings where health-care facilities rely primarily on diesel-fuelled generators, health systems—despite the overall cost savings of solar power—remain accustomed to low-capital-expenditure generators and pay-as-needed fuel purchases (sometimes financed through patient fees) and struggle to budget appropriately for more periodic costs associated with solar operation and maintenance.^2^, ^41^

There is no one-size-fits-all approach to operation and maintenance, and the most appropriate mechanism must be identified according to the local context. This principle is well illustrated by one of the largest health-care facility electrification programmes currently under implementation at the country level. In India, the partnership between health-sector authorities and the SELCO Foundation embeds operation and maintenance models that integrate system design, financing, government ownership, local enterprise capacity, digital monitoring, and proactive service agreements.^42^ Such operation and maintenance models are tailored to the ecosystem maturity, determined by three factors: geography (terrain, access, connectivity, and disaster risk), public health system (governance, fund availability, human resources, etc), and presence of suitable local energy enterprises—with weaker contexts receiving more support in terms of budget and human resources. SELCO Foundation operations are digitised through a remote monitoring platform, which enables real-time logging by health staff to communicate energy system malfunctioning. Design choices contribute to lowering the operation and maintenance burden—eg, by separating essential loads from non-essential ones, and planning for spare parts and routine checks by trained staff.^42^

## Policy options

### Improve data collection, analysis, and sharing through standardised indicators

Monitoring is an integral component of policy development and evaluation. Effective management is not possible without measurement, and systematic and periodic monitoring is essential for the design, implementation, and adaptation of sound policies. Within this framework, regular assessment of the status and quality of electricity supply—and of electricity-dependent services—in health-care facilities is crucial for guiding investments and strengthening accountability. Some surveys that have included questions on electricity access are the Service Provision Assessment, the Service Availability and Readiness Assessment, the Emergency Obstetric and Newborn Care, the Service Delivery Indicators, and the Health Resources and Services Availability Monitoring System.^2^ However, these questions are often limited in scope and relatively inconsistent across surveys. The surveys often require facilities to report only basic metrics of electricity availability or reliability, and, in most cases, such data are not yet routinely collected, analysed, or integrated into health management information systems or national health surveys. Routine collection and public availability of energy data at health-care facilities are essential to avoid duplication of efforts, enable more targeted analyses, and maximise synergies in addressing key energy and health system needs. Integrating electricity-related indicators with other health-related monitoring platforms, such as the WHO/UNICEF Joint Monitoring Programme for Water Supply, Sanitation and Hygiene, can leverage synergies to reduce costs and simultaneously increase opportunities for policy effects.

**Figure 2:**
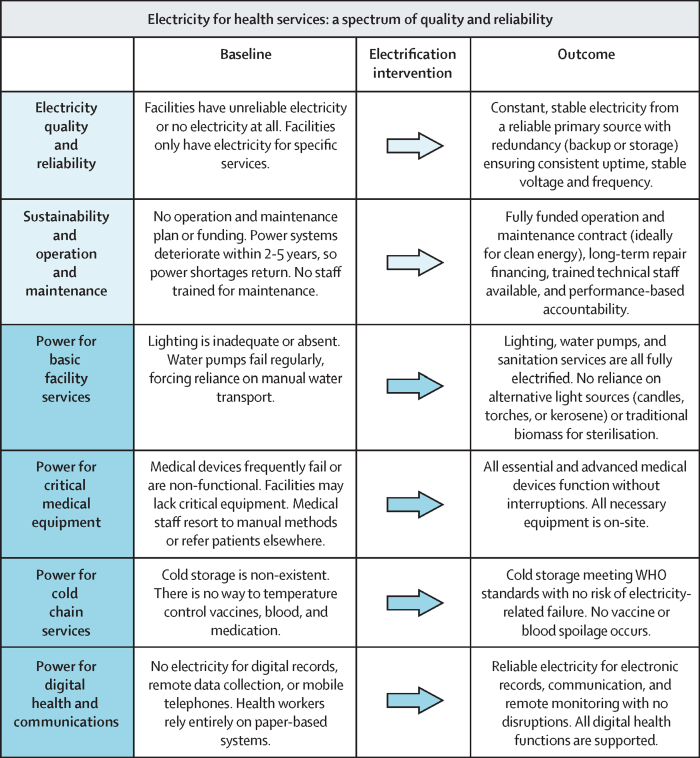
Electricity for health services: a spectrum of quality and reliability

Periodic collection of standardised (or at least harmonised) energy indicators is crucial to track the status of electricity-enabled services in health-care facilities and to guide policy making, identify priorities, increase accountability,^43^ and unlock the possibility for academic researchers to more rigorously study the potential pathways illustrated in figure 1. Examples of key constructs to track include systems-level aspects, such as electricity quality and reliability and operation and maintenance sustainability (shown in light blue in figure 2), and service-level constructs, such as power for basic facility services, critical medical equipment, cold chain services, and digital health and communications (shown in dark blue in figure 2). For each of these elements, several specific indicators could be tracked to measure the state of electrification and electricity-dependent services (table 2). For example, to characterise the electrical quality and reliability available at a health-care facility, when possible, facilities could report specific indicators such as uptime (a combination of outage incidence and outage duration), voltage fluctuations (eg, an increase or decrease of 10% of 120, 220, or 230 volts),^44^ and frequency fluctuations (eg, an increase or decrease of 5% of 50 or 60 Hz).^45^ These indicators align with energy sector metrics, facilitating collaboration between health departments and energy agencies responsible for improving electricity access and quality. To characterise operation and maintenance sustainability, facilities could track the availability of funding for future needs and the amount spent on operation and maintenance and the frequency of maintenance in the past year.

**Table 2:** Examples of possible indicators, data collection, and monitoring procedure to measure state of electrification and electrified services for health-care facilities

	**Indicator**	**Digital monitoring**	**Self-report based monitoring**
**Electricity quality and reliability**			
Uptime	Percentage of time electricity is available (uptime)	Smart meters or remote sensors continuously track power availability	Facility self-reports outages (eg, logs frequency and duration of blackouts per month)
Voltage and frequency stability	Percentage of time voltage and frequency remains within safe limits (±X% of rated voltage; ±X% of rated frequency)	Remote sensors track fluctuations, voltage spikes, and brownouts	Staff logs when medical devices are unavailable or fail due to power issues
Backup power effectiveness	Percentage of outages where backup system engages within X seconds	Automated logging of generator set or battery switch-over times using IoT sensors	Staff logs how often and how quickly backup power engages after outages
**Sustainability and operation and maintenance**			
Availability of long-term operation and maintenance funding	Existence of a funded long-term (eg, 5–10 year) operation and maintenance plan	Earmarked operation and maintenance fund disbursements tied to automated verification of energy service or maintenance performance (and relevant contracts)	Facility administrators report whether they have a maintenance budget
Budget share for operation and maintenance	Percentage of annual budget allocated for power system maintenance	Audits of health facility financial data	Survey facility managers report on maintenance spending over the past year
Maintenance frequency	Number of times system maintenance and repairs conducted in past year	Digitised record of maintenance services	Health staff log when and how often maintenance and repairs are conducted
**Basic facility services**			
Lighting uptime	Percentage of night time hours with functional lighting	Automated logs from smart lighting and battery systems	Health workers report how often they use alternative lighting (candles, torches, etc)
Availability of piped water	Availability of piped water at facility	Records or receipts of piped water system installation	Health workers report presence of piped water at facility
**Critical medical equipment**			
Equipment functionality	Percentage of time key medical devices (as appropriate for facility type) are functional	Remote monitoring systems on medical devices track when used	Health workers record devices breakdowns due to power failures
Equipment availability	Number of critical medical devices available and number out of service due to power issues	Equipment inventory systems track active *vs* non-functional devices compared with standard requirements	Facility maintenance logs track device downtime and cause of failures
**Cold chain and sterilisation**			
Vaccine fridge temperature	Percentage of time vaccine refrigerators maintain required temperature	Remote temperature loggers track continuous temperature data	Manual logbooks track when refrigerators lose power for extended periods
Vaccine dose spoilage	Number of vaccine doses spoiled due to power issues	Cold chain monitoring platforms integrate temperature logs and vaccine loss data	Staff record vaccine spoilage incidents linked to power failures
Sterilisation	Presence of an electric autoclave	Equipment inventory system includes record of electric autoclave being used	Manual logbooks track presence and use of electric autoclave for sterilisation
**Digital health and communications**			
Connectivity uptime	Percentage of time internet and telecommunications are functional	Network connectivity sensors track internet access times	Staff reports when internet or phone lines are unavailable due to power

IoT=internet of things.

Establishing the precise thresholds associated with each indicator will require further research. Moreover, although this section focuses on electricity-related indicators, further characterising their links with health-related indicators will be important. Health-system-related data, which are often routinely collected at facilities through health management information systems, will be crucial for the analysis of electricity-health linkages at health-care facilities. In this context, health-care facility electrification programmes should systematically assess not only the long-term functionality of facility energy systems but also the impacts of electricity—and of electricity reliability—on health service delivery and outcomes.

Table 2 provides examples of how facilities might operationalise data collection for each indicator using either digital tools or self-reported methods. Although digital tools can be used for data collection, each indicator could be self-reported. Low-cost, real-time sensors offer a promising example of how digital tools can improve data accuracy and reduce reporting burdens on facility staff. Instead of generic information on energy supply and quality, for example, based on health facility staff recalling or tracking whether there were no power interruptions of more than 2 hours per day during the past 7 days,^46^ sensors can provide detailed data from the facilities they are installed in. These data could be processed with relatively low-cost, low-latency, and high-volume data analysis systems to provide facility technicians or energy system managers with actionable insights to address issues in real-time. Digital monitoring via low-cost sensors has expanded rapidly across multiple sectors, with Global System for Mobile Communications-enabled handpump loggers, water-filter and cookstove monitors, sanitation-use devices, and electricity-use sensors, showing that automated, high-frequency data collection is now technically mature and widely deployed within and beyond health-care settings.^47^, ^48^, ^49^, ^50^, ^51^

### Increase investment in energy infrastructure for health-care facilities

Health is a fundamental human right, and electricity access in health-care facilities is crucial to achieving universal access to quality health services. Health-care electrification through renewable energies effectively addresses multiple Sustainable Development Goals (SDGs), including SDG 3 on health, SDG 7 on clean energy, and SDG 13 on climate.

Investments in decentralised solar systems provide climate resilience, energy independence, and cost savings compared with diesel-fuel-based options and should therefore be considered strategic investments. More importantly than the economic aspect, the main return on investment of energy interventions in health-care facilities lies in the lives saved.

Despite the dramatic benefits of health-care facility electrification, this is not yet considered a priority by many organisations that support renewable energy and climate action, and funding aimed at supporting health-care facility electrification is still inadequate.^52^ Grants are a particularly important form of financing, especially in low-resource and fragile, conflict-affected, and vulnerable settings, where the health sector is already facing multiple challenges in dramatic financial situations. Beyond grants, in certain contexts, innovative, scalable financial models could leverage scarce public and philanthropic funds and mobilise private sector capital. Table 3 illustrates the main categories for decentralised solar electrification models of health-care facilities, from the traditional capital-expenses-focused approach, in which the public sector owns and maintains the solar system, to the energy-services-based approach, in which a local service provider, such as a private sector company, instals, owns, and maintains the energy system, providing electricity to the health-care facility as a paid service over a multiyear period (eg, 10–15 years).

**Table 3:** Main health-care facility solar electrification models

	**Traditional capital-expenses-based model**	**Hybrid model(s)**	**Energy-service-based model**
Solar electrical system ownership	Public sector ownership	Public sector ownership	Developer ownership (with potential transfer to public sector after a certain period of operation)
Operation and maintenance responsibilities	Operation and maintenance through public institutions	Operation and maintenance outsourced (partially or totally) to local service providers (private sector companies, non-governmental organisations, etc)	Developer or local service provider

Development-focused financing institutions and governments in LMICs can incentivise private sector involvement, for example, through energy service models, by offering risk mitigation instruments to cover the risk of non-payment from public institutions. Other tools could include first-loss guarantees, investment aggregation support mechanisms, and value-added tax exemptions for equipment imports, among other instruments. Catalytic capital could also crowd-in health technology solution developers and their financiers,^53^, ^54^ stimulating the innovation needed to widen accessibility to cost-effective health treatment. By strategically facilitating public–private cooperation, these mechanisms can maximise the effect of low resources and accelerate progress towards universal health coverage. Although the private sector can play an important role (eg, as energy service providers), electrification of health-care facilities represents an important public health intervention, and as such, governments have the key responsibility to accelerate progress and ensure that the benefits are for all, including the most vulnerable populations.

Climate finance could also play an important role in accelerating health-care electrification. Between 2009 and 2019, only 5% of climate adaptation funds were used for health activities despite the effect of climate change on health.^55^ Accounting for health benefits could serve as an important indicator to drive climate investments. Such accounting of health benefits could complement development-related or climate-related support mechanisms, such as outcome bonds^56^ or carbon credits that could be earned by offsetting diesel emissions at facilities that rely on diesel generators for primary or backup power.

Other market-based mechanisms are emerging, such as the distributed renewable energy certificate or peace renewable energy certificate.^57^ Carbon credits and these certificates could allow projects, especially if aggregating the effect on multiple facilities, to sell carbon offsets in global or regional markets and can be used to finance renewable energy projects in health-care facilities. Results-based and outcome-based financing—in combination with digital monitoring platforms—could further attract investors, increase accountability and mitigate risks, by linking funding to measurable energy (and health) outcomes.^41^, ^58^, ^59^

Effective coordination among stakeholders, including in designing and testing new delivery models, is crucial to unlocking economies of scale and ensuring long-term sustainability. However, although it is important to explore innovative delivery models that can address current gaps and challenges, this should not serve as an excuse to delay financial support and investments for health-care facility electrification. There remains an urgent need to immediately increase financial support from governments, donors, development partners, and financing institutions, including grant funding for the most vulnerable and fragile contexts.

### Prioritise equitable health facility electrification at the national and international level

Accelerating the electrification of health-care facilities requires not only funding but also sustained political commitment. Governments can make progress towards SDG 3 and SDG 7 by integrating health-care electrification targets into national and subnational electrification and health plans, as well as other relevant development policies. This integrated approach enables a comprehensive assessment of the political, developmental, and economic trade-offs involved in allocating resources for health-care facility electrification. Governments can further promote an equitable focus on remote and other underserved communities through tailored plans, supportive policies, and regulatory frameworks, including subsidies, tax incentives, and streamlined approval processes. Ensuring that health-care facility electrification efforts reach underserved communities with the greatest need is crucial to protect the health of the most vulnerable populations, advance health equity, and contribute to socioeconomic development.

International organisations play a key role in prioritising health-care facility electrification, in partnership with local governments, non-governmental organisations, and other relevant actors. The continued consolidation of global alliances focused on health-care facility electrification—involving organisations such as WHO, UNICEF, Gavi, the World Bank, African Development Bank, or the Africa Centres for Disease Control and Prevention—together with strengthened cross-sectoral cooperation at the country level, will be crucial to sustaining long-term attention to equitable progress, with particular emphasis on low-resource and fragile settings affected by conflict and violence.

### Strengthen coordination between health and energy sectors and between governments, practitioners, and researchers

Increased coordination and collaboration between the energy and health sectors is essential to accelerate health-care facility electrification. Such coordination is required not only at the international and national levels but also among subnational institutions, non-governmental organisations, service providers, and researchers. Effective coordination is necessary to avoid duplication of efforts, maximise the effect of interventions, and leverage synergies. This coordination might include joint energy–health needs assessments, identification of priority facilities to be electrified through grid extension, or off-grid solutions, pooled funding mechanisms, long-term operation and maintenance arrangements, e-waste management, the training of technicians, etc.

At the international level, the Health and Energy Platform of Action, hosted by WHO, and multiple other actors, such as the Global Energy Alliance for People and Planet, Sustainable Energy for All, and the International Federation of Red Cross and Red Crescent Societies, are promoting strengthened collaboration between the health and energy sectors, including the integration of technical, economic, and social sustainability considerations into health-care facility electrification planning.^2^, ^60^ However, success depends heavily also on effective coordination at the country level. The Global Heatmap of health facility electrification and its associated database, managed by Sustainable Energy for All, indicate that multiple electrification initiatives operate within several countries, varying widely in scope and scale, and requiring enhanced in-country coordination among institutional actors and development partners.^61^

At the national level, health ministries and rural electrification agencies can work together to prioritise health facilities for electrification. At subnational levels, successful models of coordination can be developed with the involvement of local communities. At the service provider level, entities responsible for water supply, sanitation, medical devices, and waste management services at health-care facilities need to work with energy actors to ensure that their electricity demands are considered, and ensure that health-care facilities are provided with the necessary power-dependent medical devices and appliances once they receive access to electricity. Effective coordination mechanisms might include multisectoral working groups, joint planning and budgeting processes, and knowledge-sharing platforms to foster collaboration. To avoid duplication of efforts and strengthen coordination, governments, development partners, and donors could benefit from a centralised country dashboard displaying both the status of electrification of each health-care facility and planned or ongoing projects supported by different actors.

Safe management of e-waste, including batteries and solar modules, at the end of energy system life cycles is also essential. Through the adoption and enforcement of appropriate regulations and procurement requirements, such as the use of quality-verified products, extended producer responsibility schemes with financed take-back and collection targets, and the engagement of audited recyclers with worker protection standards, health-care electrification efforts can ensure net benefits while protecting environment and people.^62^, ^63^

Since ensuring operation and maintenance for solar systems is a common need, actors involved in health-care facility electrification programmes should coordinate on the implementation of operation and maintenance frameworks. At the same time, development partners involved in health-care facility electrification should share information about their projects at the facility level, leveraging digital data and remote monitoring platforms. This holistic approach would ensure reliable and up-to-date information on energy status and functionality of different systems, and would promote transparency and accountability.

### Build local capacities

Leveraging local capacity to instal, operate, and maintain energy systems and equipment is essential to enhance the effectiveness and long-term sustainability of health-care facility electrification programmes, including those based on decentralised solar energy systems. In this context, training and capacity building of the local workforce play a crucial role. Strengthening local public institutions supports the design, implementation, and management of electrification programmes. Training of local technicians helps ensure sustained system functionality, and adequate training of health-care facility staff is crucial for troubleshooting common problems, undertaking preventive maintenance, and adhering to safety protocols. Engaging local energy companies in installation, operation, and maintenance services has also proven effective in ensuring the sustainability of energy systems in health-care facilities, including in humanitarian settings.^26^ Leveraging health-care facility electrification efforts to build local capacity contributes to job creation and socioeconomic development and can indirectly support further community electrification efforts.

Strengthening capacity within relevant institutions, including ministries of health and energy, is crucial for effective programme planning, implementation, and management. Strengthening capacity at the ministry level involves building durable institutional functions, such as in planning, technical oversight, financing, coordination, and accountability, which allow health-care electrification programmes to be designed, financed, and sustained at scale.

When feasible, leveraging virtual training and artificial intelligence for on-demand, language-appropriate training and maintenance support could provide technicians with specific information or instructions on a broad range of technical challenges on systems and devices.^64^ Training health-care professionals on the basic operation of energy systems and how to identify and report technical issues can speed troubleshooting when issues arise.^42^ Building capacity in the proper use of electricity-dependent medical equipment can also avoid incorrect usage that sometimes causes equipment failure. Use of electronic medical record systems, digital health and telemedicine, and how to access internet-based resources to improve the quality of care that they deliver are other examples of areas that are enabled by electricity access and that require adequate health staff training. Overall, capacity building efforts should be tailored to local needs and contexts and be a key part of health system strengthening efforts.

### Develop and deploy biomedical equipment suitable for harsh conditions

Without power-dependent medical devices and appliances, access to electricity alone cannot deliver the intended health benefits. As described earlier in this Series paper, health-care facilities require a broad range of medical equipment and appliances to provide the services expected at their level of the health-care referral pyramid. Currently, most medical equipment used in low-resource settings is imported from high-income countries. Although up to 70% of the medical equpment in sub-Saharan Africa is donated, only 10–30% of donated equpment becomes operational.^20^, ^65^ The reasons for which donated medical devices become non-functional include lack of training of local users, lack of maintenance, unavailability of material and resources required for correct functioning of device, and inadequate characteristics of a donated device in respect of the local working environment.^66^

There is a disparity between the context in which the devices are expected to function, and the context in which they do, including in terms of power stability.^20^ Although the long-term goal is to upgrade electrical systems to provide reliable, high-quality electricity, designing medical devices that are more suitable to low-resource environments, and can, for example, tolerate larger fluctuations in frequency and voltage, is another important focus. Furthermore, energy efficiency should also be considered in equipment procurement and energy systems planning, since energy-efficient equipment reduces the energy demand and costs, including the size of decentralised energy systems to be installed.

Large-scale procurement or prepurchase agreements could create market incentives for manufacturers to develop equipment appropriate for the harsh electrical and environmental conditions of low-resource settings. The design and field testing of this new equipment, however, require data on current electrical and environmental conditions and user resources and needs. A successful example of this market-shaping approach is represented by the solar direct-drive vaccine refrigerators and freezers that were designed specifically for the energy constraints and operational needs of low-resource settings.^67^ Developers were able to transition from the collection of power system data and equipment design to product quality standards development and market transformation, such that solar direct-drive refrigerators are now being deployed in the most remote facilities, although some local maintenance challenges remain. The decreasing costs of photovoltaic modules and batteries are allowing facilities to expand power capacity to power not only the refrigerator, but also entire health centres (panel).

Panel **Examples of health–energy collaboration efforts**

**Integrating power and connectivity**

In Zambia, WHO, UNICEF, and the Global Fund to fight AIDS, Tuberculosis, and Malaria are collaborating with the local authorities to support electricity access and connectivity in rural health facilities.^68^ The project will assess the effect and cost-effectiveness of multiple technology and delivery model solutions, with the aim of accelerating cross-sectoral coordination and leveraging synergies.

**Facility–community energy service model**

In Uganda, the World Bank's Electricity Access Scale-up Project is supporting long-term service agreements with energy service companies for electrifying public institutions (including schools and health facilities) with standalone solar systems.^69^ Ministries will make quarterly performance-based payments to energy service companies based on the fulfilment of predetermined key performance indicators.

**Cross-sector collaboration to leverage multiple resources**

In Sierra Leone, the Ministry of Health is collaborating with SEforALL, WHO, and other partners to pull resources for coordinated efforts. Collaborations include the ongoing Healthcare Electrification Project, which aims to electrify 25 primary health-care facilities and 17 government hospitals through solar power.^70^

**Public sector and non-governmental organisation partnership**

In India, national and state health departments and the SELCO Foundation are solarising 25 000 primary health centres, with states covering 50–60% of the capital expenditures and SELCO providing the remainder.^71^ Ensuring long-term operation and maintenance is a priority—models range from relying on local non-governmental organisations with facility or government funds to requiring the creation of 24/7 service centres.

**Local and international collaboration and research**

In the Democratic Republic of the Congo, local non-governmental organisation the Research Center for Humanitarian Aid (RCHA) is coordinating with the national electricity regulator, North Kivu's provincial health department, a local implementation partner, and two research universities (University of California, Berkeley and Duke University) to electrify facilities while doing research.^22^ RCHA's partnership has enabled the remote monitoring of over 25 facilities to track uptime and system performance and has fostered research on the health effects of electrification.

**From device-specific solutions to full facility solarisation**

WHO, GAVI, and UNICEF, with technical support from the SELCO Foundation, are working with the governments of Ethiopia, Uganda, Zambia, and Pakistan to electrify 1300 health-care facilities.^34^ The initiative builds on the cold chain equipment optimisation platform, but instead of supporting only the deployment of small solar panels tailored to the solar refrigerator needs, it supports the deployment of bigger solar electrification systems able to power all essential medical devices and key appliances in health-care facilities.

**Integrating solar electrification and provision of power-dependent medical devices**

In the Democratic Republic of the Congo, WHO is working with the Ministry of Public Health, Hygiene, and Social Welfare to electrify approximately 100 health-care facilities with solar power systems, while simultaneously equipping them with life-saving, power-dependent medical devices. This integrated approach aims to ensure that electrification efforts translate into immediate health benefits.

Overall, it is crucial to facilitate the design and production of medical devices that are suitable for harsh conditions—devices that are user-friendly, simple, robust, energy-efficient, and require low maintenance—and at the same time ensure their long-term operation and maintenance. Additionally, it is essential that every health-care facility electrification programme is coordinated with interventions to provide power-dependent medical devices and appliances, and staff training, to ensure that electricity access translates into real service delivery and improved health outcomes.

## Conclusion

Reliable, affordable, sustainable, and equitable electricity access in health-care facilities is fundamental for universal health coverage and should be recognised as a global development priority. Decentralised renewable energy systems present a key opportunity to rapidly expand access to reliable electricity, particularly for remote facilities where grid connection is unavailable, and as a backup for facilities with unreliable energy.

Although the private sector has an important role to play in scaling up electrification, the public sector must take the lead in prioritising and financing these efforts to ensure universal access to health care, which is a human right. Financial support from donors and development partners is crucial, and health-care facility electrification should be prioritised within broader energy and climate support funding mechanisms. Long-term operation and maintenance, and appropriate funding mechanisms to support these costs, are also key for energy sustainability.

Systematic data collection of standardised indicators—preferably through digital systems and real-time monitoring—along with increased investments, prioritisation of equity, strengthened coordination, and access to biomedical devices suitable for harsh conditions, are all essential for advancing health care electrification efforts and maximising impact, and avoiding siloed approaches. Future research is needed to provide more detailed causal estimates of how different dimensions of electricity (eg, reliability or quality) affect various aspects of service delivery and health outcomes. A better understanding of these relationships could enable the refinement and validation of health-care facility-based power quality and reliability indicators, inform tailored interventions, and support the development of more robust biomedical equipment.

Given that energy is necessary but not sufficient in itself to improve health outcomes, health-care facility electrification efforts will be most successful when they are integrated into broader health system strengthening strategies that also invest in training staff, providing electricity-dependent medical equipment, and ensuring the availability of necessary supplies, vaccines, and medications. Achieving universal access to reliable electricity in health-care facilities is both a moral imperative and a strategic investment that will pay dividends in improved health outcomes for all.

### Contributors

## Declaration of interests

We declare no competing interests.

## References

[1] WHO (2015).

[2] WHO, World Bank, Sustainable Energy for All, International Renewable Energy Agency (2023).

[3] WHO (2020).

[4] Stanturf JA, Goodrick SL, Warren ML, Charnley S, Stegall CM (2015). Social vulnerability and ebola virus disease in rural Liberia. PLoS One.

[5] Khogali A, Ahmed A, Ibrahim M (2022). Building power-ful health systems: the impacts of electrification on health outcomes in LMICs. Psychol Health Med.

[6] UN General Assembly (Dec 21, 2023). Resolution ‘Sustainable, safe and universal water, sanitation, hygiene, waste and electricity services in health-care facilities’, 78/130, adopted by the General Assembly on 18 December 2023. https://docs.un.org/en/A/RES/78/130?utm.

[7] Rokicki S, Mwesigwa B, Waiswa P, Cohen J (2021). Impact of solar light and electricity on the quality and timeliness of maternity care: a stepped-wedge cluster-randomized trial in Uganda. Glob Health Sci Pract.

[8] Chen YJ, Chindarkar N, Xiao Y (2019). Effect of reliable electricity on health facilities, health information, and child and maternal health services utilization: evidence from rural Gujarat, India. J Health Popul Nutr.

[9] Apenteng BA, Opoku ST, Ansong D, Akowuah EA, Afriyie-Gyawu E (2018). The effect of power outages on in-facility mortality in healthcare facilities: evidence from Ghana. Glob Public Health.

[10] Duke T, Pulsan F, Panauwe D (2021). Solar-powered oxygen, quality improvement and child pneumonia deaths: a large-scale effectiveness study. Arch Dis Child.

[11] Mian Q, Huang Y, Conroy A, Opoka R, Namasopo S, Hawkes M (2019). Solar-powered oxygen delivery to treat childhood pneumonia in low-resource settings: a randomised controlled non-inferiority trial and cost-effectiveness study. Lancet Glob Health.

[12] Duke T, Wandi F, Jonathan M (2008). Improved oxygen systems for childhood pneumonia: a multihospital effectiveness study in Papua New Guinea. Lancet.

[13] Huang Y, Mian Q, Conradi N (2021). Estimated cost-effectiveness of solar-powered oxygen delivery for pneumonia in young children in low-resource settings. JAMA Netw Open.

[14] WHO (2024).

[15] Economist Impact, UNICEF (May 28, 2024). Powering progress: measuring the benefits of investing in energy resilience for healthcare, education and water. https://impact.economist.com/sustainability/resilience-and-adaptation/sustainable-energy-in-emerging-economies.

[16] World Bank Live (Jan 28, 2025). Advancing Africa's energy future at the Mission 300 Africa Energy Summit. https://live.worldbank.org/en/event/2025/mission-300-africa-energy-summit.

[17] WHO Database on electrification of health-care facilities. https://www.who.int/data/gho/data/themes/database-on-electrification-of-health-care-facilities.

[18] Sustainable Energy For All Global heatmap. https://www.seforall.org/programmes/powering-healthcare-hub/global-heatmap.

[19] Diaconu K, Chen Y-F, Cummins C (2017). Methods for medical device and equipment procurement and prioritization within low- and middle income countries: findings of a systematic literature review. Global Health.

[20] WHO (2010).

[21] Hinman R, Jónsson P, Kreyche T (Dec 4, 2019). https://web.archive.org/web/20250317135818/http://power.2to8.cc/Power_Quality_Challenges_In_Low_Resource_Settings_2019.pdf.

[22] Miles SB, Mughuma JM, Moner-Girona M, Kammen DM, Kwong LH (2025). The power of health in the Democratic Republic of the Congo: electricity quality and reliability in medical facilities of North Kivu Province. Appl Energy.

[23] Franco A, Shaker M, Kalubi D, Hostettler S (2017). A review of sustainable energy access and technologies for healthcare facilities in the Global South. Sustain Energy Technol Assess.

[24] Chawla S, Kurani S, Wren SM (2018). Electricity and generator availability in LMIC hospitals: improving access to safe surgery. J Surg Res.

[25] Grafham O, Lahn G (December, 2018). The costs of fuelling humanitarian aid. Moving Energy Initiative. https://www.chathamhouse.org/sites/default/files/publications/research/2018-12-10-Costs-Humanitarian-Aid2.pdf.

[26] Archimi J, Javeri S, Soto K (2025).

[27] Sandwell P, Gibson M, Fohgrub T (2021).

[28] Farquharson D, Jaramillo P, Samaras C (2018). Sustainability implications of electricity outages in sub-Saharan Africa. Nat Sustain.

[29] Al-akori A, Ansari D, Cader C, Brahim W, Blechinger P (2023). Conflict, health, and electricity: an empirical assessment of the electrification of healthcare facilities in Yemen. Energy Res Soc Sci.

[30] Cronk R, Bartram J (2018). Environmental conditions in health care facilities in low- and middle-income countries: coverage and inequalities. Int J Hyg Environ Health.

[31] Reuland F, Behnke N, Cronk R (2020). Energy access in Malawian healthcare facilities: consequences for health service delivery and environmental health conditions. Health Policy Plan.

[32] Trotter PA (2021). From silos to systems: enabling off-grid electrification of healthcare facilities, households, and businesses in sub-Saharan Africa. One Earth.

[33] Moner-Girona M, Kakoulaki G, Falchetta G, Weiss DJ, Taylor N (2021). Achieving universal electrification of rural healthcare facilities in sub-Saharan Africa with decentralized renewable energy technologies. Joule.

[34] Gavi, the Vaccine Alliance (Oct 11, 2024). Ethiopia Ministry of Health, Gavi, UNICEF and WHO launch Health Facility Solar Electrification (HFSE) initiative to enhance primary health care services. https://www.gavi.org/news/media-room/ethiopia-ministry-health-gavi-unicef-and-who-launch-hfse.

[35] International Renewable Energy Agency, SELCO Foundation (2025).

[36] Sustainable Energy for All (Dec 3, 2023). Climate finance for powering healthcare. https://www.seforall.org/system/files/2024-02/report-phc-climate-finance-002.pdf.

[37] WHO (June 17, 2019). Powering health: WHO brings solar energy to the health sector in Gaza. https://www.emro.who.int/fr/opt/news/powering-health-who-brings-solar-energy-to-the-health-sector-in-gaza.html.

[38] WHO (Dec 13, 2024). UN and partners join a simulation exercise in Ghana to test new ways to respond to outbreaks. https://www.who.int/news-room/feature-stories/detail/un-and-partners-join-a-simulation-exercise-in-ghana-to-test-new-ways-to-respond-to-outbreaks.

[39] SolarAid (March, 2022). Powering healthcare in Malawi. https://solar-aid.org/wp-content/uploads/2022/04/Powering-Healthcare-Malawi-March-2022.pdf.

[40] Yudhistira R, Khatiwada D, Sanchez F (2022). A comparative life cycle assessment of lithium-ion and lead-acid batteries for grid energy storage. J Clean Prod.

[41] Sustainable Energy for All (2024).

[42] SELCO Foundation (2025). Powering the future—a sustainability-focused maintenance guide for decentralised solar energy in public institutions. https://selcofoundation.org/wp-content/uploads/2025/09/Powering-the-Future-OM-Guide.pdf.

[43] Anderson DM, Bartram J, Tantum LK (2025). With a new United Nations resolution on water, sanitation, hygiene, electricity, and waste in healthcare facilities, it is time for a logical framing and consistent vocabulary. PLOS Sustain Transform.

[44] Stepka E (Oct 26, 2020). ANSI C84.1-2020: electric power systems voltage ratings (60 Hz). https://blog.ansi.org/2020/10/ansi-c84-1-2020-electric-voltage-ratings-60/.

[45] WHO (March 1, 2020). How to develop and publish a PQS product performance specification. https://extranet.who.int/prequal/sites/default/files/document_files/MHP_RPQ_PQT_VAX_PQS_SOP_001%20How%20to%20develop%20and%20publish%20a%20PQS%20product%20performance%20specification.pdf.

[46] WHO (2015).

[47] Rom A, Günther I, Borofsky Y (2020). Using sensors to measure technology adoption in the social sciences. Dev Eng.

[48] Hartvigsson E, Ahlgren EO (2018). Comparison of load profiles in a mini-grid: assessment of performance metrics using measured and interview-based data. Energy Sustain Dev.

[49] Ramanathan T, Ramanathan N, Mohanty J, Rehman IH, Graham E, Ramanathan V (2017). Wireless sensors linked to climate financing for globally affordable clean cooking. Nat Clim Chang.

[50] Thomas EA, Barstow CK, Rosa G, Majorin F, Clasen T (2013). Use of remotely reporting electronic sensors for assessing use of water filters and cookstoves in Rwanda. Environ Sci Technol.

[51] Nagel C, Beach J, Iribagiza C, Thomas EA (2015). Evaluating cellular instrumentation on rural handpumps to improve service delivery—a longitudinal study in rural Rwanda. Environ Sci Technol.

[52] IEA, IRENA, UNSD, World Bank, WHO (2024).

[53] De Maria C, Díaz Lantada A, Jämsä T, Pecchia L, Ahluwalia A (2022). Biomedical engineering in low- and middle-income settings: analysis of current state, challenges and best practices. Health Technol.

[54] WHO (2017). Global atlas of medical devices. https://iris.who.int/handle/10665/255181.

[55] Alcayna T, O'Donnell D, Chandaria S (2023). How much bilateral and multilateral climate adaptation finance is targeting the health sector? A scoping review of official development assistance data between 2009–2019. PLOS Glob Public Health.

[56] World Bank Group (January, 2025). Impact investing with the World Bank. https://thedocs.worldbank.org/en/doc/6513fbb4895a8c3a69cf450309d7e708-0340022025/original/World-Bank-Foundation-and-Development-Partner-Opportunities-Updated-January-2025.pdf.

[57] Kammen D, Yoshikawa H, Yamaguchi K (2023).

[58] Elahi R, Srinivasan R, Mukurazhizha T (January, 2020). Increasing human capital by electrifying health centers and schools through off-grid solar solutions. https://hdl.handle.net/10986/33276.

[59] World Bank (July 5, 2022). Digital monitoring, reporting, and verification systems and their application in future carbon markets. https://hdl.handle.net/10986/37622.

[60] WHO Health and energy platform of action. https://www.who.int/initiatives/health-and-energy-platform-of-action.

[61] Sustainable Energy For All Powering healthcare hub discover insights. https://www.seforall.org/programmes/powering-healthcare-hub/discover-insights.

[62] WHO (2021).

[63] International Renewable Energy Agency, IEA Photovoltaic Power Systems Programme (2016).

[64] WHO (2024).

[65] Marks IH, Thomas H, Bakhet M, Fitzgerald E (2019). Medical equipment donation in low-resource settings: a review of the literature and guidelines for surgery and anaesthesia in low-income and middle-income countries. BMJ Glob Health.

[66] WHO (July 29, 2024). Medical device donations: considerations for solicitation and provision, second edition. https://www.who.int/publications/i/item/9789240093621.

[67] Gavi The Vaccine Alliance (May, 2021). Market shaping roadmap ice-lined (ILR) and solar direct drive (SDD) refrigerators/freezers. Public summary. https://www.gavi.org/sites/default/files/document/ice-lined-and-solar-direct-drive-refrigerators-freezers-public-summarypdf.pdf.

[68] The Global Fund (April, 2025). Zambia. Digital health case study. https://www.theglobalfund.org/media/w4gdfjob/publication_zambia-digital-health_casestudy_en.pdf.

[69] Republic of Uganda Ministry of Education and Sports (Jan 28, 2025). Bidding document for the procurement of non-consultancy services. Supply, installation, commissioning, operation and maintenance of solar photovoltaic systems, electrical works and lighting for forty-five (45) seed secondary schools in three (3) lots. https://www.education.go.ug/wp-content/uploads/2025/01/Bidding-Documents_EASP_MoES_3-Lots_Published.pdf.

[70] Ministry of Health (Sierra Leone) (Nov 18, 2024). Massive boost for electrification of health facilities in Sierra Leone as MoH and partners commission 25 PHUs with solar power. https://mohs.gov.sl/massive-boost-for-electrification-of-health-facilities-in-sierra-leone-as-moh-and-partners-commission-25-phus-with-solar-power/.

[71] ClimaHealth (2023). ‘Energy for Health’ initiative for renewable energy at 25,000 primary health facilities in India. https://climahealth.info/resource-library/energy-for-health-initiative-for-renewable-energy-at-25000-primary-health-facilities-in-india/#:~:text=Approach,such%20facilities%20have%20been%20powered.

